# Interleukin-6 upregulates extracellular matrix gene expression and transforming growth factor β1 activity of tendon progenitor cells

**DOI:** 10.1186/s12891-023-07047-9

**Published:** 2023-11-22

**Authors:** Nadine Altmann, Charles Bowlby, Haley Coughlin, Zarah Belacic, Stasia Sullivan, Sushmitha Durgam

**Affiliations:** Department of Veterinary Clinical Sciences, College of Veterinary Medicine, The Ohio State University, Columbus, OH USA

**Keywords:** Interleukin-6, Superficial digital flexor tendon, TGFβ1, ECM mRNA gene expression, Matrix metalloproteinases

## Abstract

**Background:**

Prolonged inflammation during tendon healing and poor intrinsic healing capacity of tendon are causal factors associated with tendon structural and functional degeneration. Tendon cells, consisting of mature tenocytes and tendon progenitor cells (TPC) function to maintain tendon structure via extracellular matrix (ECM) synthesis. Tendon cells can succumb to tissue cytokine/chemokine alterations during healing and consequently contribute to tendon degeneration. Interleukin-(IL-)1β, IL-6 and TNFα are key cytokines upregulated in injured tendons; the specific effects of IL-6 on flexor tendon-derived TPC have not been discerned.

**Methods:**

Passage 3 equine superficial digital flexor tendon (SDFT)-derived TPC were isolated from 6 horses. IL-6 impact on the viability (MMT assay with 0, 1, 5 and 10 ng/mL concentrations), migration (scratch motility assay at 0, 10ng/mL concentration) of TPC in monolayer culture were assessed. IL-6 effect on tendon ECM and chondrogenic gene expression (qRT-PCR), TGFβ1 gene expression and activity (ELISA), and MMP-1, -3 and − 13 gene expression of TPC was evaluated.

**Results:**

IL-6 decreased TPC viability and migration. IL-6 treatment at 10 ng/mL significantly up-regulated TGFβ1 gene expression (6.3-fold; *p* = 0.01) in TPC, and significantly increased the TGFβ1 concentration in cell culture supernates. IL-6 (at 10 ng/mL) significantly up-regulated both tendon ECM (COL1A1:5.3-fold, COL3A1:5.4-fold, COMP 5.5-fold) and chondrogenic (COL2A1:3.9-fold, ACAN:6.2-fold, SOX9:4.8-fold) mRNA expression in TPC. Addition of SB431542, a TGFβ1 receptor inhibitor, to TPC in the presence of IL-6, attenuated the up-regulated tendon ECM and chondrogenic genes.

**Conclusion:**

IL-6 alters TPC phenotype during in vitro monolayer culture. Pro- and anti-inflammatory roles of IL-6 have been implicated on tendon healing. Our findings demonstrate that IL-6 induces TGFβ1 activity in TPC and affects the basal TPC phenotype (as evidenced via increased tendon ECM and chondrogenic gene expressions). Further investigation of this biological link may serve as a foundation for therapeutic strategies that modulate IL-6 to enhance tendon healing.

**Supplementary Information:**

The online version contains supplementary material available at 10.1186/s12891-023-07047-9.

## Background

Achilles and superficial digital flexor tendinopathies from overuse due to athletic activities in people and horses, respectively, are debilitating and common. Disrupted collagen fibers and increased ground substance within the tendons reduces the mechanical strength of tissue, and consequently predisposes the individual to repeat tendon injuries (up to 50–60%) [1–3]. Persistent and prolonged inflammation during tendon healing combined with the poor intrinsic healing capacity of tendons are key causal factors responsible for the degenerative tissue characteristics [4–8]. The inflammatory and regulatory processes responsible for tendon degeneration, and consequently, reduced tissue mechanical strength is yet to be elucidated. Cytokines such as interleukin-1β (IL-1β), IL-6 and tumor necrosis factor-α (TNFα) are up-regulated and in turn induce inflammatory mediators cyclooxygenase-2 (COX-2), prostaglandin E2 (PGE2), and collagenases, matrix metalloproteinases (MMP) such as MMP-1, -3 and − 13; all involved in tendon degeneration [7–9].

The cellular fraction of tendons, consisting of mature tenocytes and tendon progenitor cells (TPC), function to maintain tendon structure via extracellular matrix (ECM) synthesis and turnover [10, 11]. Histological characterization of tendinopathy lesions demonstrate that tendon cells undergo cellular ‘rounding’ and develop chondrocyte-like characteristics with concurrent increase in tissue glycosaminoglycan (GAG) concentrations [12, 13]. Tendon cells are susceptible to changes in local cytokines/chemokine concentrations and in turn are implicated in tendon degeneration [14–16]. In-vivo studies have documented increased intratendinous IL-6 concentrations in naturally occurring human tendinitis and experimental rat models of tendinitis [17–19]. Katsma et al., demonstrated that exogenous IL-6 injections down-regulated MMP-1 and collagen type I transcript levels in healing rat Achilles tendon injuries [17]. In-vitro, IL-6 down-regulated critical tendon phenotype markers, Scleraxis and tenomodulin in rat Achilles tendon-derived TPC [20]. Taken together, while elevated tendon IL-6 concentrations during healing have been attributed to pro-resolving effects as well as towards tendon degeneration, the associated biological mechanisms are largely unknown.

The objective of this study was to evaluate the impact of IL-6, a key cytokine encountered in tendon degeneration, on equine SDFT-derived TPC. We investigated the effects of IL-6 on TPC viability, migration (via standard scratch assay), and tendon ECM, chondrogenic and MMP gene expressions. Additionally, we investigated IL-6 effects on TPC profibrotic growth factor TGFβ1 activity, by culturing TPC supplemented with IL-6 in the presence of TGFβ receptor (ALK-4, − 5, −7) inhibitor, SB431542.

## Methods

### TPC isolation and culture

TPC were isolated from forelimb SDFT of 6 (mean age 4.2 ± 2.2 years) adult horses using protocols previously described [11, 21–23]. TPC were characterized via equine MSC surface marker expression profile (CD 44^+^, CD29^+^, CD90^+^, and CD45^-^) and in vitro trilineage differentiation as previously described (Supplemental Fig. 1; 21, 22, 23). Briefly, intact forelimb SDFT were harvested from young adult horses euthanized for reasons unrelated to musculoskeletal disease. Mid-metacarpal SDFT specimens (1–2 cm length), free of peritendinous tissue, were diced into 2–3 mm^3^ pieces and digested in 0.15% collagenase (Worthington) in DMEM supplemented with 2% fetal bovine serum (Gemini Biomedical) at 37° C for 16 h. The released cells were isolated by filtration and centrifugation, and the cells were seeded at 2000 cells/cm^2^ in monolayer cultures in high-glucose DMEM supplemented with 10% fetal bovine serum (FBS), 37.5 µg/mL of ascorbic acid, 300 µg of L-glutamine/mL 100 U of sodium penicillin/mL, and 100 µg of streptomycin sulfate/mL (basal medium). These cells were seeded onto cell culture flasks and incubated at 37° C with 5% CO_2_ to enable colony formation. Medium was replaced every 3 days. Once discernible colonies were formed (> 200 cells/colony), the cells were detached with 0.05% trypsin EDTA. Cell numbers were calculated by counting an aliquot of the resulting suspension using a hemocytometer and an inverted light microscope [21, 23, 24]. Trypan blue dye exclusion was used to assess cell viability. The primary cell isolates were reseeded at 5 × 10^3^ cells/cm^2^ and passaged twice at 80–90% confluence to expand cell numbers and enrich for progenitor cells. Passage 3 TPC (henceforth referred to as TPC) were used for all subsequent experiments.

### TPC viability

TPC were plated at 3 × 10^3^ cells/cm^2^ in basal medium in 96-well plates. After 24 h of culture, low serum medium (2% FBS) supplemented with IL-6 (at 1, 5, 10 ng/mL; R&D Systems) was added and incubated for 3 days. Three replicate wells for each IL-6 concentration were used to measure the cell numbers via a mitochondrial metabolic assay (Cell Titer MTT 96 aqueous one solution cell proliferation assay, Promega) which was used in accordance with the manufacturer’s instructions [24]. In brief, 20 µL of the assay reagent containing tetrazolium was added into each well of the 96-well plate containing 100 µL of fresh media and incubated at 37° C for 2.5 h. Absorbance was measured at 490 nm in a microplate reader (Tecan™ Infinite 200 PRO plate reader) to detect concentrations of the metabolic product, formazan. The absorbance of basal media alone was used as the ‘blank’ and subtracted from all experimental sample optical density readings. The mean value was calculated from replicate wells to provide a single data point. The optical density values from plated TPC from each horse were reported.

### ‘Scratch assay’ assessment of TPC motility

TPC were cultured as monolayers in basal medium, as described above, in 100 mm culture dishes, until near confluence. The monolayers were then transferred to low serum medium (2% FBS) alone or with 1, 5 or 10 ng/mL IL-6. A 20 uL sterile pipette tip was used to score a narrow grove in the cell monolayers, which approximately yields a 1.0 mm wide ‘scratch defect’ [24]. Cell migration into the defect was measured 24 h later on photomicrographs acquired with a 5x objective and Leica DMIL microscope and DFC 320 digital camera (Leica Microsystems, Leica Application Suite- LAS- version 4.2). An average measurement from twenty-five individual measurements of the remaining defect width was obtained using ImageJ (https://imagej.nih.gov) “Set Scale” and “Measure” tools (measured by three investigators blinded to the treatment conditions).

### Monolayer culture for transcriptional assays

TPC were cultured as monolayers in basal medium, as described above, in 50 cm^2^ culture dishes, until 80% confluence. The monolayers were then transferred to low serum medium (2% FBS) alone or with 10 ng/mL IL-6. Fresh medium was replaced every 48 h. The cultures were maintained for 3 days. Additionally, TPC were also cultured with 0 or 2 μm TGFβ type I receptor (ALK-4, − 5, −7) inhibitor, SB431542 (Selleckchem; 13), in the presence of 0 and 10 ng/mL IL-6 for 3 days. Then, the monolayers were detached from the culture dishes with a cell scraper, centrifuged at 800 g for 10 min, medium removed, snap frozen in liquid nitrogen and stored in − 80 C until RNA isolation. Transcriptional assays were conducted from n = 5 TPC.

### RNA isolation and quantitative RT-PCR

Total RNA was isolated using a previously described protocol [21, 25]. RNA yields and A260/280 values are compiled in Supplemental Table 1. The samples were homogenized in guanidinium thiocyanate phenol-chloroform solution reagent (TRIzol, Invitrogen) according to the manufacturer’s suggested protocol. The resultant pellet was purified using RNeasy silica columns that included on-column DNase digestion. The concentration of RNA was determined by measuring the absorbance at 260 nM (A260) and 320 nM (A320) in NanoDrop One/One® (Thermo Fisher Scientific). One µg of RNA from each sample was reverse-transcribed (Superscript IV, Invitrogen) using oligo (dT) primers. Equine gene-specific primers were designed from published sequences in Genbank and using ClustalW multiple sequence alignment (Table1; available at http://www.ebi.ac.uk). Primer specificity was confirmed by cloning and sequencing the amplicons during optimization experiments, as previously described [23, 24]. PCR amplifications were catalyzed by Taq DNA polymerase (ABI QuantStudio 3™, Thermo Fisher Scientific) in the presence of SYBR green. Relative gene expression was quantified using the 2-ΔΔCT method, normalized to expression of the reference gene, elongation factor-1α (EF1α) [26].

**Table 1:** Primers for SYBRgreen RT-qPCR

Gene	Accession Number		Sequence	Amplicon (bp)
Col I	NC_009154	S A	5’ GAA AAC ATC CCA GCC AAG AA 5’ GAT TGC CAG TCT CCT CAT CC	231
Col III	AW261123	S A	5’ AGG GGA CCT GGT TAC TGC TT 5’ TCT CTG GGT TGG GAC AGT CT	215
COMP	NM_001081856	S A	5’ TCA TGT GGA AGC AGA TGG AG 5’ TAG GAA CCA GCG GTA GGA TG	223
Sox9	XM_023452130	S A	5’ GAA CGC ACA TCA AGA CGG AG 5’ CTG GTG GTC TGT GTA GTC GT	304
Col II	NM_001081764.1	S A	5’ AGC AGG AAT TTG GTG TGG AC 5’ TCT GCC CAG TTC AGG TCT CT	223
Aggrecan	XM_023650277.1	S A	5’ GAC GCC GAG AGC AGG TGT 5’ AAG AAG TTG TCG GGC TGG TT	202
MMP-1	AF148882.1	S A	5’ GGT GAA GGA AGG TCA AGT TCT GAT 5’ AGT CTT CTA CTT TGG AAA AGA GCT TCT C	232
MMP-3	GDHK01064470	S A	5’ GGC AAC GTA GAG CTG AGT AAA GCC 5’ CAA CGG ATA GGC TGA GCA CGC	286
MMP-13	AF034087.1	S A	5’ AAG CCA CTT TGT GCT TCC TGA T 5’ GGA TCG CAT TTG TCT GGT GTT	220
TGFβ1	AF175709.1	S A	5’ ATC AAC GGG TTC AGT TCC AG 5’ CGC AGC AGT TCT TCT CTG TG	244
EF1-α	NM_001081781.1	S A	5’ CCC GGA CAC AGA GAC TTC AT 5’ AGC ATG TTG TCA CCA TTC CA	328

### Medium TGFβ1 concentration

TGFβ1 concentration in the respective culture medium (three replicates per treatment per horse) were determined with a commercially available ELISA kits (Quantikine, R&D Systems, Minneapolis, MN) in accordance with the manufacturer’s protocol. Briefly, the collected medium samples from 0, 1, 5 or 10 ng/mL IL-6, and control and 10 ng/mL IL-6 cultured with 0 or 2 μm SB431542 were individually combined with TGFβ1 conjugate. The samples were washed and incubated with a substrate solution, and optical density was measured with a microplate reader set at 450 nm. The optical densities of standards with known concentrations were used to generate a standard curve from which the concentration of the individual samples was determined. All standards and samples were analyzed in duplicate.

### Statistical analysis

Normal distribution of data was assessed by Shapiro-Wilks’s test using SigmaPlot 14 software (Systat, San Jose, CA). The data were representative of at least five independent experiments, each done in triplicate. All results are expressed as the mean ± standard deviation or median (range). One-way ANOVA or the non-parametric equivalent, Kruskal-Wallis test on ranks was used to compare the data. Post hoc comparisons for the detection of statistically significant differences between TPC treated with 0, 1, 5, 10 ng/mL IL-6 (proliferation, motility, TGFβ1 gene expression and medium TGFβ1 concentration), and basal media (Control), 10 ng/mL IL-6 (IL-6), 2 μm TGFβ type I receptor (ALK-4, − 5, −7) inhibitor SB431542 (I), IL-6 + I was conducted with Holm-Sidak or Tukey’s method. Differences were considered statistically significant at *P* ≤ 0.05.

## Results

### TPC viability

**Fig. 1:**
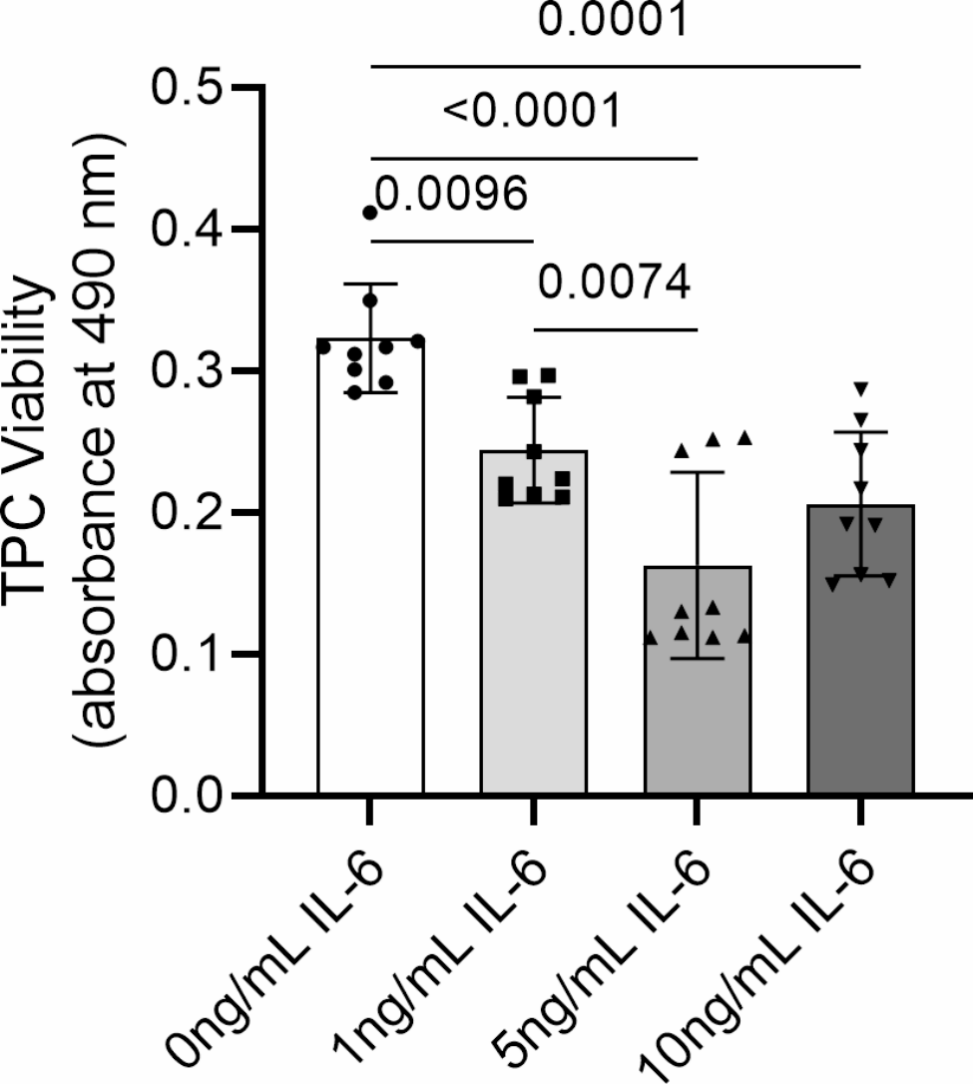
TPC viability measured via MTT assay after 24 h of monolayer culture in basal medium supplemented with 0, 1, 5 and 10 ng/mL IL-6. The y-axis denotes absorbance readings measured at 490 nm. Bars and errors represent mean ± SD

After 3 days of monolayer culture, mean ± SD optical density values reflecting TPC viability demonstrated that 1, 5 and 10 ng/mL IL-6 significantly reduced TPC viability by 1.3 ± 0.156-fold (*p* = 0.0096), 2.03 ± 0.175 (*p* < 0.0001) and 1.65 ± 0.0.154-fold (*p* = 0.0001), respectively (Fig. 1).

### Scratch assay assessment of motility

**Fig. 2:**
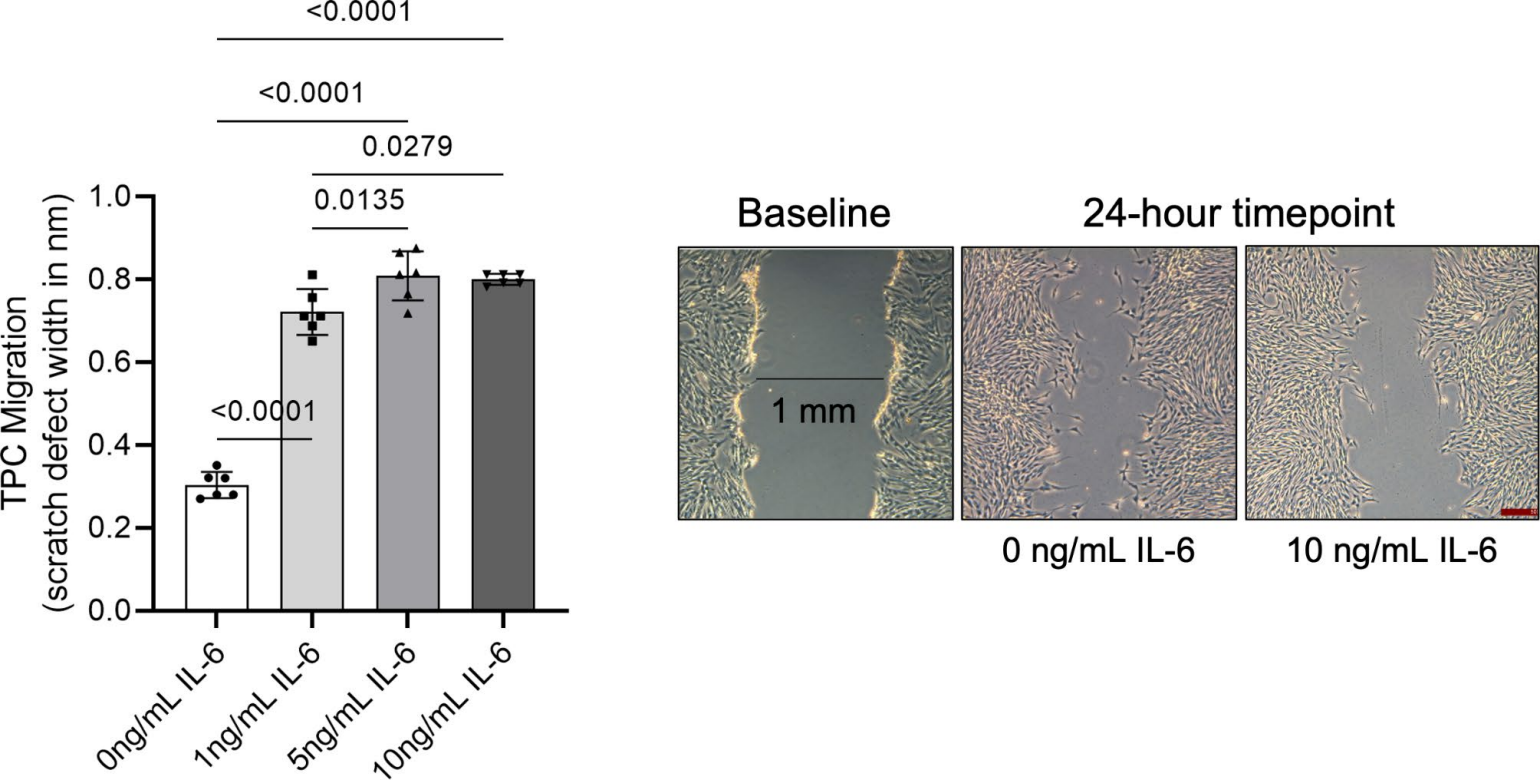
TPC migration assessed via ‘scratch assay’ measured as the width of the defect remaining after 24 h of monolayer culture in basal medium supplemented with 0, 1, 5 and 10 ng/mL IL-6. Bars and errors represent mean ± SD of “scratch” defect width after 24 h. Supporting 5X photomicrographs of TPC monolayers at baseline, and at 24-hour timepoint with 0 and 10 ng/mL IL-6. Scale bar represents 200 microns

TPC monolayers in basal (2% FBS) medium rapidly migrated across the “scratch” defect, and by 24 h the mean width of the defect was significantly (7 ± 0.15-fold; *p* = 0.02) decreased from the initial width of the defect. In contrast, all three concentrations (1, 5 and 10 ng/mL) of IL-6 significantly (*p* < 0.0001) impaired TPC migration across the scratch defect (Fig. 2). In addition, 5 (*p* = 0.0135) and 10 (*p* = 0.0279) ng/mL of IL-6 treatments significantly decreased TPC migration across the scratch defect to a greater extent than 1 ng/mL IL-6 treatment.

### TGFβ1 gene expression and culture medium TGFβ1 concentration

**Fig. 3:**
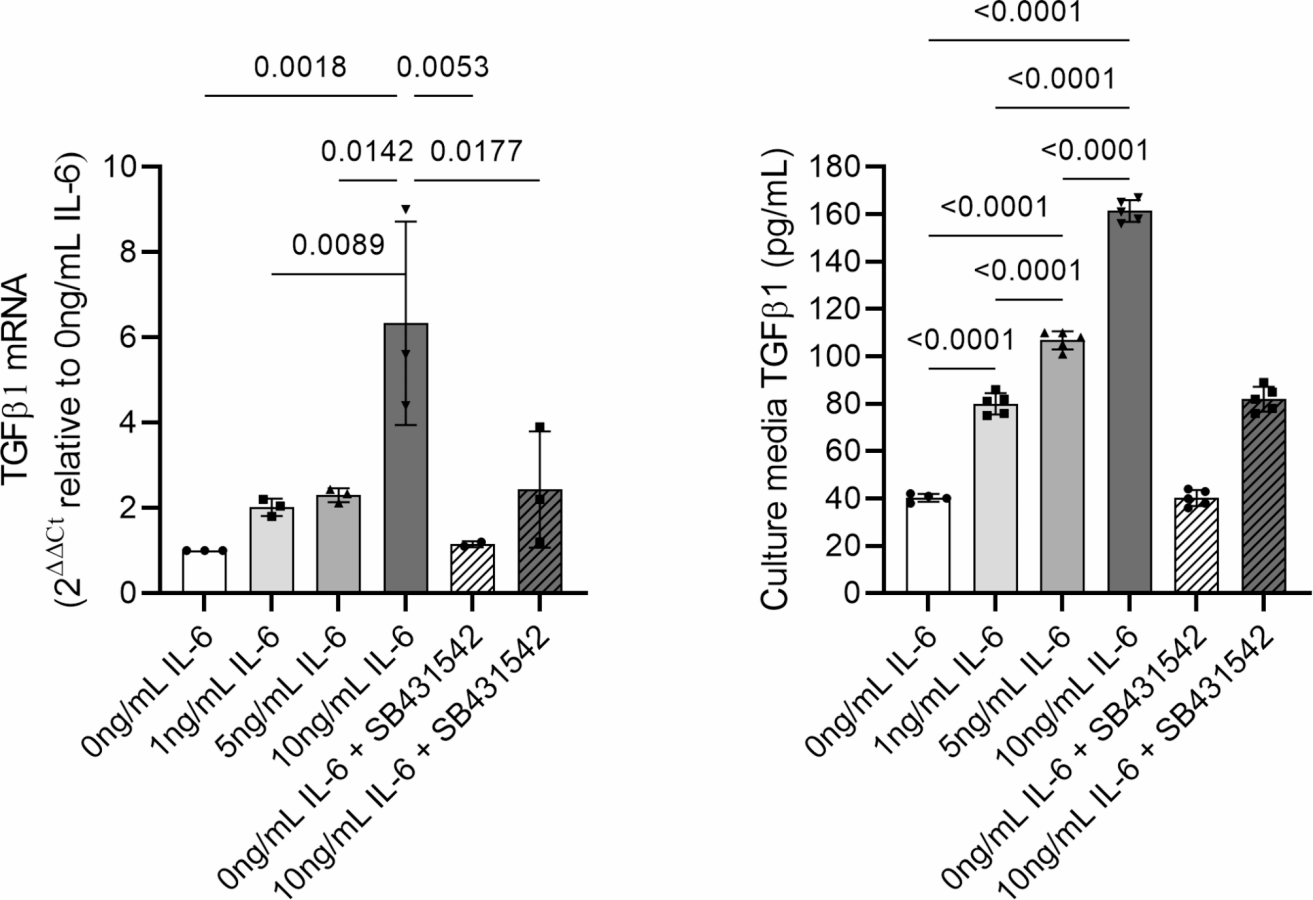
**(A)** TGFβ1 mRNA and **(B)** ELISA quantification of TGFβ1 concentration measured in TPC monolayer culture and culture media, respectively, (mean ± SD) after 72 h of culture with 0, 1, 5 and 10 ng/mL IL-6. TGFβ1 mRNA and concentration were also measured in TPC in basal medium and 10 ng/mL IL-6 supplemented with 2 μm TGFβ type I receptor (ALK-4, − 5, −7) inhibitor SB431542. Bars and errors represent mean ± SD of TGFβ1 mRNA and concentration (pg/mL)

TGFβ1 mRNA (6.3 ± 2.3-fold) level was significantly (*p* = 0.032) upregulated with 10ng/mL IL-6 compared to untreated control, whereas 1 and 5 ng/mL IL-6 did not significantly change TGFβ1 mRNA (Fig. 3A). Culture medium TGFβ1 concentration was significantly increased with 1, 5 and 10 ng/mL IL-6 concentrations. TGFβ1 concentrations in the control and 10 ng/mL IL-6 + SB431542 were not significantly different from each other (Fig. 3B).

### Tendon ECM gene expression

**Fig. 4:**
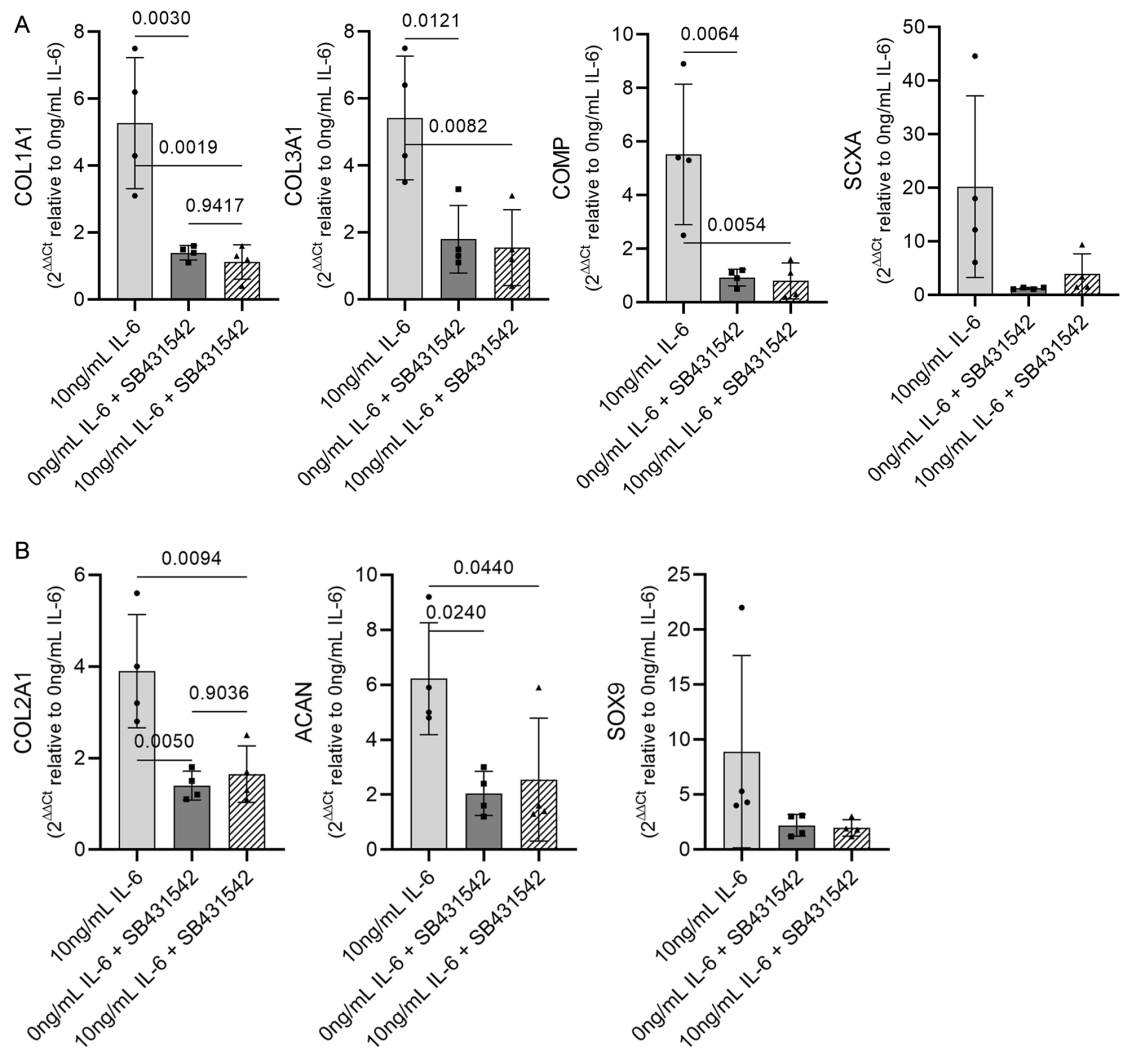
**(A)** Tendon ECM and **(B)** Chondrogenic gene expression of TPC monolayers cultured in basal medium with 10 ng/mL IL-6, basal medium with 2 μm TGFβ type I receptor (ALK-4, − 5, −7) inhibitor SB431542, and 10 ng/mL IL-6 + SB431542 for 72 h. Data represents mean ± SD of fold change (normalized to housekeeping gene, EF1α) from respective values obtained from TPC monolayer cultures in basal medium alone (represented via dotted line in all graphs)

*COL1A1* (5.3 ± 2.0-fold, *p* < 0.001), *COL3A1* (5.4 ± 1.8-fold, *p* = 0.004), and *COMP* (5.5 ± 2.6-fold, *p* = 0.002) mRNA levels in TPC were significantly upregulated with 10 ng/mL IL-6 compared to control (Fig. 4A). TGFβ inhibitor, SB431452 attenuated IL-6-induced mRNA upregulation. TGFβ inhibitor did not affect the basal TPC tendon ECM gene expression.

### Chondrogenic gene expression

*COL2A1* (3.9 ± 1.28-fold, *p* = 0.002), *ACAN* (6.2-fold ± 2.0, *p* = 0.007) and *SOX9* (4.8-fold ± 20.2, *p* = 0.003) mRNA levels in TPC were significantly upregulated with 10 ng/mL IL-6 compared to untreated control (Fig. 4B). Consistent with tendon ECM gene expression, TGFβ inhibitor, SB431452 attenuated IL-6-induced chondrogenic mRNA upregulation. TGFβ inhibitor did not affect the basal TPC chondrogenic gene expression.

### Matrix metalloproteinase (MMP) gene expression

IL-6 significantly upregulated *MMP-1* (7.5 ± 3.2-fold, *p* = 0.01), *MMP-3* (21 ± 4.5-fold, *p* < 0.01), and *MMP-13* (4.2 ± 2.8- fold, *p* = 0.041) mRNA levels in TPC compared to untreated controls.

## Discussion

Interleukin-6 is a multifactorial cytokine that regulates inflammation, angiogenesis, as well as immunomodulation during injury development and healing of several tissues, through interactions with tissue-resident cells and the ECM [14, 17, 19]. In tendon, IL-6 maintains collagen fiber size, organization and consequently, tendon mechanical strength during development and healing as determined with transgenic murine models [19]. While continuous/prolonged exercise and chronic injury lead to increased IL-6 levels within and in tendon periphery; the specific effects of IL-6 on TPC that maintain tenocytes and in turn tendon ECM are largely undetermined. Our results demonstrate that IL-6 reduced the viability and migration of equine SDFT-derived TPC in monolayer culture. In this study, IL-6 upregulated both tendon ECM and chondrogenic gene expression in equine SDFT-derived TPC. IL-6 upregulated TGFβ1 gene expression as well as TGFβ1 secretion into the culture medium. Finally, the addition of TGFβ1 inhibitor, SB431542 attenuated IL-6-induced gene upregulations, without affecting the basal ECM gene expression of TPC in monolayer culture.

We observed inhibitory effects of IL-6 on equine SDFT-derived TPC viability and migration properties. Previous studies have reported both pro- and anti-proliferative and migratory effects of IL-6 on fibroblast cells. Chen et al. reported a significant increase in rat Achilles tendon-derived TPC proliferation with 1, 10 and 100 ng/mL of IL-6; however, this was only noted at day 5 of monolayer culture, and was not apparent at day 1 and 3 of IL-6 exposure [20]. In murine and human myoblasts, lower concentrations of IL-6 (10 and 100 pg/mL) promoted cell proliferation, whereas IL-6 at 10 ng/mL concentration inhibited proliferation and promoted myogenic differentiation [27]. The divergent effects of IL-6 were both attributed to STAT3 signaling pathway, a pathway critical for cell survival and proliferation. Collectively, IL-6 has displayed variable influences on tissue fibroblast proliferation depending on their lineage, exposure duration, and if obtained from normal or diseased tissues. It is important to note that these studies do not consider the tissue ECM, and further investigations focused on dissecting IL-6 effects on TPC/tenocyte viability, proliferation and migration, interplay with ECM, and the associated mechanisms during in vivo tendon healing are warranted.

Tendon progenitor cells within injured tendon exhibit increased chondrogenic capacity and are implicated in chondroid degenerative lesions within injured/healing tendons [11, 13, 23]. This study identified that IL-6 induced profibrotic growth factor TGFβ1 activity, and significantly increased both tendon ECM and chondrogenic gene expressions. This is in contrast to Chen et al., where IL-6 treatment of rat Achilles tendon-derived TPC decreased tendon ECM gene expression [20]. We evaluated IL-6 effects on TGFβ1 activity, since TGFβ1 is upregulated in injured tendon, and supraphysiological levels of TGFβ1 within tendons is associated with fibrosis and chondroid degeneration [13, 28]. Transforming growth factor-β signaling via Smad pathway regulates tenogenic transcription factor, Scleraxis, essential for tendon homeostasis and collagen type I gene expression [29–31]. Smad pathway also modulates the activity of critical chondrogenic transcription factor SOX9 during chondrogenesis and induces transcription of cartilage ECM molecules such as collagen type II and aggrecan [28, 32]. In vivo, IL-6 injection in the peritendinous region inhibited collagen types I and III gene expressions in rat Achilles tendon [17]; whereas, increased collagen synthesis was noted in human Achilles tendon [33]. These disparate effects of IL-6 suggest species differences, and notable interactions with ECM and mechanical loading that are lacking in in vitro cell culture studies and highlights the need for follow up investigations to validate the in vitro findings. This study along with others indicate that IL-6 induces TGFβ1 activity in fibroblastic cell types [18, 34, 35], however the related causal mechanisms, and impact on TPC biosynthesis warrants further work.

This in vitro study has several limitations. In addition to the known subject-related variability between TPC populations and relatively low sample size (n = 6), IL-6 effects were only evaluated in monolayer culture and does not account for tendon ECM and mechanical loading present in vivo. IL-6 influence on TPC in vitro at up to 10ng/mL concentration was assessed only at a single time point (72 h after treatment). Obtaining gene expression and ECM protein biosynthesis measurements at a series of timepoints following treatment would have provided the temporal effects of IL-6. Given the differential in vitro and in vivo effects of IL-6 on TPC [36–38], validating these in vitro results with in vivo studies is necessary. In both experimental and naturally occurring tendon injuries, IL-6 and MMP gene and protein expressions are concomitantly increased [36–38]. Our results found that IL-6 increased MMP-1 and MMP-3 gene expressions by 37% (*p* = 0.06) and 54% (*p* = 0.02), respectively; however, corresponding changes in culture medium supernate enzyme concentrations were not investigated in this study.

Pro- and anti-inflammatory roles of IL-6 on tendon healing have been documented [38, 39], and is dependent on the local inflammatory environment and tendon healing phase. Moreover, tendon tissue degeneration during healing is a complex process that encompasses orchestrated interplay of several inflammatory/regulatory cytokines, chemokines, and growth factors. Our findings demonstrate that IL-6 induces TGFβ1 activity in TPC and affects the basal TPC phenotype (as evidenced via increased tendon ECM and chondrogenic gene expressions). Further investigation of this biological link may serve as a foundation for therapeutic strategies that modulate IL-6 to enhance tendon healing. Although the mechanisms resulting in aberrant TPC phenotypes and consequent tendon degeneration are complex, results of this study augment current evidence that TPC can function as target cells to develop therapeutic strategies for enhancing tendon healing. Given that IL-6 alters TPC phenotype, the efficacy of novel and existing cell-based and biological therapies aiming to enhance tendon healing can be determined in part by measuring their effect on local IL-6 concentrations.

## Conclusions

These results demonstrate that IL-6 alters the phenotype of TPC in in vitro monolayer culture. While TGFβ signaling is critical for lineage-specific transcription factor expression in several mesenchymally-derived tissues, it is also involved in scar formation/fibrosis in several healing tissues and linked to chondrogenic metaplasia and heterotopic ossification in healing tendon. Pro- and anti-inflammatory roles of IL-6 have been implicated on tendon healing. Our findings demonstrate that IL-6 induces TGFβ1 activity in TPC and affects the basal TPC phenotype (as evidenced via increased tendon ECM and chondrogenic gene expressions). Further investigation of this biological link may serve as a foundation for therapeutic strategies that modulate IL-6 to enhance tendon healing.

### Electronic supplementary material

Below is the link to the electronic supplementary material.

Supplementary Material 1

## Data Availability

Datasets for all gene sequences can be found in the GENBANK repository [https://www.ncbi.nlm.nih.gov/genbank/]. The raw data sets (gene expression, MTT, and scratch test, ELISA) supporting the results of this manuscript will be made available by the corresponding author, without any reservation, to researchers upon request.

## List of Abbreviations

IL: Interleukin
TPC: Tendon progenitor cells
SDFT: Superficial digital flexor tendon
ECM: Extracellular matrix
TGFβ1: Transforming growth factor β1
MMP: Matrix metalloproteinase
COMP: Cartilage oligomeric matrix protein
GAG: Glycosaminoglycan
DMEM: Dulbecco’s modified Eagle’s medium
FBS: Fetal bovine serum

## References

[1] O’Meara B, Bladon B, Parkin TD, Fraser B, Lischer CJ (2010). An investigation of the relationship between race performance and superficial digital flexor tendonitis in the Thoroughbred racehorse. Equine Vet J.

[2] Lam KH, Parkin TD, Riggs CM, Morgan KL (2007). Descriptive analysis of retirement of Thoroughbred racehorses due to tendon injuries at the Hong Kong Jockey Club (1992–2004). Equine Vet J.

[3] Lam KK, Parkin TD, Riggs CM, Morgan KL (2007). Evaluation of detailed training data to identify risk factors for retirement because of tendon injuries in Thoroughbred racehorses. Am J Vet Res.

[4] Birch HL, Wilson AM, Goodship AE (2008). Physical activity: does long-term, high-intensity exercise in horses result in tendon degeneration?. J Appl Physiol (1985).

[5] Goodship AE, Birch HL, Wilson AM (1994). The pathobiology and repair of tendon and ligament injury. Vet Clin North Am Equine Pract.

[6] Spiesz EM, Thorpe CT, Chaudhry S, Riley GP, Birch HL, Clegg PD (2015). Tendon extracellular matrix damage, degradation and inflammation in response to in vitro overload exercise. J Orthop Res.

[7] Thorpe CT, Chaudhry S, Lei II, Varone A, Riley GP, Birch HL (2015). Tendon overload results in alterations in cell shape and increased markers of inflammation and matrix degradation. Scand J Med Sci Sports.

[8] Mobasheri A, Shakibaei M (2013). Is tendinitis an inflammatory Disease initiated and driven by pro-inflammatory cytokines such as interleukin 1β?. Histol Histopathol.

[9] Ko JY, Wang FS, Huang HY, Wang CJ, Tseng SL, Hsu C (2008). Increased IL-1beta expression and myofibroblast recruitment in subacromial bursa is associated with rotator cuff lesions with shoulder stiffness. J Orthop Res.

[10] Kannus P (2000). Structure of the tendon connective tissue. Scand J Med Sci Sports.

[11] Bi Y, Ehirchiou D, Kilts TM, Inkson CA, Embree MC, Sonoyama W (2007). Identification of tendon stem/progenitor cells and the role of the extracellular matrix in their niche. Nat Med.

[12] Cook JL, Feller JA, Bonar SF, Khan KM (2004). Abnormal tenocyte morphology is more prevalent than collagen disruption in asymptomatic athletes’ patellar tendons. J Orthop Res.

[13] Asai S, Otsuru S, Candela ME, Cantley L, Uchibe K, Hofmann TJ (2014). Tendon progenitor cells in injured tendons have strong chondrogenic potential: the CD105-negative subpopulation induces chondrogenic degeneration. Stem Cells.

[14] Blomgran P, Blomgran R, Ernerudh J, Aspenberg P (2016). A possible link between loading, inflammation and healing: Immune cell populations during tendon healing in the rat. Sci Rep.

[15] Stolk M, Klatte-Schulz F, Schmock A, Minkwitz S, Wildemann B, Seifert M (2017). New insights into tenocyte-immune cell interplay in an in vitro model of inflammation. Sci Rep.

[16] Abraham AC, Shah SA, Golman M, Song L, Li X, Kurtaliaj I (2019). Targeting the NF-κB signaling pathway in chronic tendon Disease. Sci Transl Med.

[17] Katsma MS, Patel SH, Eldon E, Corbell KA, Shimkus KL, Fluckey JD (2017). The influence of chronic IL-6 exposure, in vivo, on rat Achilles tendon extracellular matrix. Cytokine.

[18] Kawamoto H, Iwatsuki K, Kurimoto S, Yamamoto M, Tatebe M, Morita A (2020). Interleukin-6 secretion by fibroblasts in carpal tunnel syndrome patients is associated with trigger finger and inhibited by tranilast. Muscle Nerve.

[19] Lin TW, Cardenas L, Glaser DL, Soslowsky LJ (2006). Tendon healing in interleukin-4 and interleukin-6 knockout mice. J Biomech.

[20] Chen S, Deng G, Li K, Zheng H, Wang G, Yu B (2018). Interleukin-6 promotes proliferation but inhibits tenogenic differentiation via the Janus Kinase/Signal Transducers and activators of transcription 3 (JAK/STAT3) pathway in Tendon-derived stem cells. Med Sci Monit.

[21] Durgam S, Schuster B, Cymerman A, Stewart A, Stewart M (2016). Differential Adhesion Selection for Enrichment of Tendon-derived progenitor cells during in Vitro Culture. Tissue Eng Part C Methods.

[22] Williamson KA, Lee KJ, Humphreys WJ, Comerford EJ, Clegg PD, Canty-Laird EG (2015). Restricted differentiation potential of progenitor cell populations obtained from the equine superficial digital flexor tendon (SDFT). J Orthop Res.

[23] Durgam SS, Stewart AA, Sivaguru M, Wagoner Johnson AJ, Stewart MC (2016). Tendon-derived progenitor cells improve healing of collagenase-induced flexor tendinitis. J Orthop Res.

[24] Durgam SS, Altmann NN, Coughlin HE, Rollins A, Hostnik LD (2019). Insulin enhances the in vitro osteogenic capacity of Flexor Tendon-Derived Progenitor cells. Stem Cells Int.

[25] Durgam SS, Stewart AA, Pondenis HC, Yates AC, Evans RB, Stewart MC (2012). Responses of equine tendon- and bone marrow-derived cells to monolayer expansion with fibroblast growth factor-2 and sequential culture with pulverized tendon and insulin-like growth factor-I. Am J Vet Res.

[26] Livak KJ, Schmittgen TD (2001). Analysis of relative gene expression data using real-time quantitative PCR and the 2(-Delta Delta C(T)) method. Methods.

[27] Steyn PJ, Dzobo K, Smith RI, Myburgh KH. Interleukin-6 induces myogenic differentiation via JAK2-STAT3 signaling in mouse C2C12 myoblast cell line and primary human myoblasts. Int J Mol Sci. 2019;20(21). 10.3390/ijms20215273 PMC6862063 31652937

[28] Sakabe T, Sakai K, Maeda T, Sunaga A, Furuta N, Schweitzer R (2018). Transcription factor scleraxis vitally contributes to progenitor lineage direction in wound healing of adult tendon in mice. J Biol Chem.

[29] Loiselle AE, Yukata K, Geary MB, Kondabolu S, Shi S, Jonason JH (2015). Development of antisense oligonucleotide (ASO) technology against Tgf-β signaling to prevent scarring during flexor tendon repair. J Orthop Res.

[30] Bagchi RA, Czubryt MP (2012). Synergistic roles of scleraxis and smads in the regulation of collagen 1α2 gene expression. Biochim Biophys Acta.

[31] Maeda T, Sakabe T, Sunaga A, Sakai K, Rivera AL, Keene DR (2011). Conversion of mechanical force into TGF-β-mediated biochemical signals. Curr Biol.

[32] Akiyama H (2008). Control of chondrogenesis by the transcription factor Sox9. Mod Rheumatol.

[33] Andersen MB, Pingel J, Kjær M, Langberg H (2011). Interleukin-6: a growth factor stimulating collagen synthesis in human tendon. J Appl Physiol (1985).

[34] Elias JA, Lentz V, Cummings PJ (1991). Transforming growth factor-beta regulation of IL-6 production by unstimulated and IL-1-stimulated human fibroblasts. J Immunol.

[35] Seong GJ, Hong S, Jung SA, Lee JJ, Lim E, Kim SJ (2009). TGF-beta-induced interleukin-6 participates in transdifferentiation of human Tenon’s fibroblasts to myofibroblasts. Mol Vis.

[36] Viganò M, Lugano G, Orfei CP, Menon A, Ragni E, Colombini A et al. Tendon cells derived from the long head of the biceps and the supraspinatus tendons of patients affected by rotator cuff tears show different expressions of inflammatory markers. Connect Tissue Res. 2020:1–10. 10.1080/03008207.2020.1816993 32921180

[37] Ueda Y, Inui A, Mifune Y, Takase F, Kataoka T, Kurosawa T (2019). Molecular changes to tendons after collagenase-induced acute tendon injury in a senescence-accelerated mouse model. BMC Musculoskelet Disord.

[38] Tarafder S, Chen E, Jun Y, Kao K, Sim KH, Back J (2017). Tendon stem/progenitor cells regulate inflammation in tendon healing via JNK and STAT3 signaling. Faseb j.

[39] Legerlotz K, Jones ER, Screen HR, Riley GP (2012). Increased expression of IL-6 family members in tendon pathology. Rheumatology (Oxford).

