# Supplementary material for: Interleukin-6 upregulates extracellular matrix gene expression and transforming growth factor β1 activity of tendon progenitor cells
Source: BMC Musculoskelet Disord. 2023 Nov 22;24:907. doi: 10.1186/s12891-023-07047-9 (PMC10664499; doi:10.1186/s12891-023-07047-9)
Supplement: Supplementary file 1 — Supplementary Material 1 [file 12891_2023_7047_MOESM1_ESM.docx]

Figure 1: (A) Representative flow cytometry composite depicting CD29+, CD44+, CD90+ and CD45- equine SDFT-derived TPC.


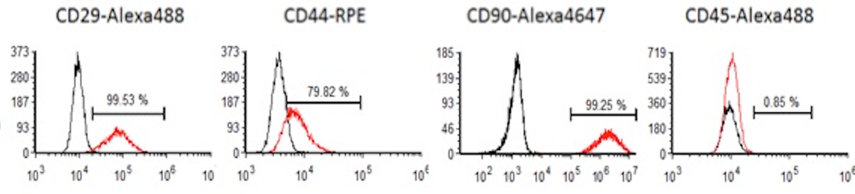


(B) Representative trilineage differentiation photomicrographs depicting adipogenesis (Oil-Red-O stained monolayers ), osteogenesis (Alizarin Red stained monolayer) and chondrogenesis (Toluidine Blue stained TPC pellets) potentials of equine SDFT-derived TPC. Bar = 100 microns


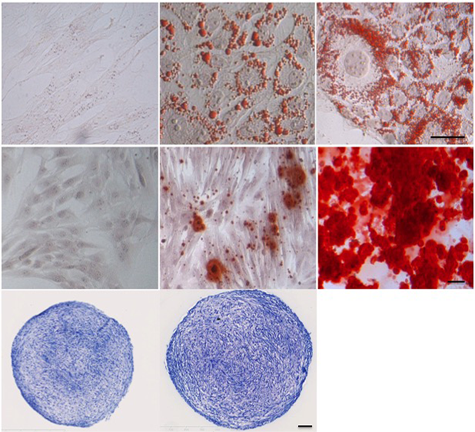


Day 14

Day 7

Day 0

Adipogenesis

Day 7

Day 14

Day 0

Osteogenesis

Day 20

Day 10

Chondrogenesis

Table 1: Nanodrop measurements of total RNA isolated in the experiments. A260/280 reading, concentrations (ng/uL) and volume used for cDNA synthesis are tabulated.

| Horse or subject number | Treatment condition | 260/280 | ng/uL RNA | Volume (uL) used for 1ug total RNA for cDNA synthesis |
| --- | --- | --- | --- | --- |
| E4 | Basal medium | 2.089 | 653.4 | 1.5 |
| E4 | Basal medium + 1ng/mL IL-6 | 2.082 | 536.5 | 1.9 |
| E4 | Basal medium + 5ng/mL IL-6 | 2.012 | 482.5 | 2.0 |
| E4 | Basal medium + 10ng/mL IL-6 | 2.112 | 337.8 | 3.0 |
| E4 | Basal medium + SB431542 | 2.012 | 358.03 | 2.8 |
| E4 | Basal medium + 10ng/mL IL-6 + SB431542 | 2.035 | 140.3 | 7.1 |
| E3 | Basal medium | 2.091 | 395.14 | 2.5 |
| E3 | Basal medium + 1ng/mL IL-6 | 2.006 | 250.2 | 4.0 |
| E3 | Basal medium + 5ng/mL IL-6 | 2.112 | 386.2 | 2.6 |
| E3 | Basal medium + 10ng/mL IL-6 | 2.021 | 440.12 | 2.5 |
| E3 | Basal medium + SB431542 | 2.006 | 348.2 | 2.87 |
| E3 | Basal medium + 10ng/mL IL-6 + SB431542 | 2.05 | 385.12 | 2.6 |
| E19 | Basal medium | 2.02 | 395.14 | 2.53 |
| E19 | Basal medium + 1ng/mL IL-6 | 2.05 | 356.0 | 2.8 |
| E19 | Basal medium + 5ng/mL IL-6 | 2.112 | 256.2 | 3.8 |
| E19 | Basal medium + 10ng/mL IL-6 | 2.00 | 337.8 | 3.0 |
| E19 | Basal medium + SB431542 | 2.012 | 358.03 | 2.8 |
| E19 | Basal medium + 10ng/mL IL-6 + SB431542 | 2.05 | 756.6 | 1.32 |
| E22 | Basal medium | 2.002 | 852 | 1.17 |
| E22 | Basal medium + 10ng/mL IL-6 | 2.112 | 747.2 | 1.34 |
| E22 | Basal medium + SB431542 | 2.012 | 135.7 | 7.37 |
| E22 | Basal medium + 10ng/mL IL-6 + SB431542 | 2.035 | 200.5 | 5.0 |
| E23 | Basal medium | 2.091 | 525.6 | 1.9 |
| E23 | Basal medium + 10ng/mL IL-6 | 2.112 | 640.2 | 1.56 |
| E23 | Basal medium + SB431542 | 2.012 | 645.7 | 1.55 |
| E23 | Basal medium + 10ng/mL IL-6 + SB431542 | 2.035 | 240.3 | 4.2 |

| Horse or subject number | Treatment condition | GAPDH | b-actin | EF1-a |
| --- | --- | --- | --- | --- |
| E4 | Basal medium | 17.5 | 21 | 13.8 |
| E4 | Basal medium + 10ng/mL IL-6 | 18.1 | 20.4 | 14.1 |
| E4 | Basal medium + SB431542 | 17.8 | 21.2 | 13.7 |
| E4 | Basal medium + 10ng/mL IL-6 + SB431542 | 18.2 | 20.6 | 14.1 |
| E3 | Basal medium | 17.8 | 20.8 | 13.7 |
| E3 | Basal medium + 10ng/mL IL-6 | 19.2 | 20.6 | 14.2 |
| E3 | Basal medium + SB431542 | 18.4 | 21.6 | 13.6 |
| E3 | Basal medium + 10ng/mL IL-6 + SB431542 | 17.8 | 22.1 | 14.1 |

Table 2: RTPCR Ct values for n=2 for housekeeping genes GAPDH, β-actin and EF1-α
